# A novel bio-mimicking, planar nano-edge microelectrode enables enhanced long-term neural recording

**DOI:** 10.1038/srep34553

**Published:** 2016-10-12

**Authors:** Pierre Wijdenes, Hasan Ali, Ryden Armstrong, Wali Zaidi, Colin Dalton, Naweed I. Syed

**Affiliations:** 1 Biomedical Engineering Graduate Program, University of Calgary, Calgary, Canada; 2 Department of Electrical and Computer Engineering, University of Calgary, Calgary, Canada; 3 Hotchkiss Brain Institute, University of Calgary, Calgary, Canada; 4 Alberta Children’s Hospital Research Institute, University of Calgary, Calgary, Canada

## Abstract

Our inability to accurately monitor individual neurons and their synaptic activity precludes fundamental understanding of brain function under normal and various pathological conditions. However, recent breakthroughs in micro- and nano-scale fabrication processes have advanced the development of neuro-electronic hybrid technology. Among such devices are three-dimensional and planar electrodes, offering the advantages of either high fidelity or longer-term recordings respectively. Here, we present the next generation of planar microelectrode arrays with “nano-edges” that enable long-term (≥1 month) and high fidelity recordings at a resolution 15 times higher than traditional planar electrodes. This novel technology enables better understanding of brain function and offers a tremendous opportunity towards the development of future bionic hybrids and drug discovery devices.

Various neuronal activity recording and stimulating devices enable cellular exploration^1^,^2^,^3^ using micro- and nano-devices^4^. A multitude of penetrating and non-penetrating nanopillar electrodes^5^,^6^,^7^, carbon nanotube electrodes^8^, mushroom-shaped protruding microelectrodes^9^, planar microelectrode arrays (MEAs) etc. have been used to record neuronal activities. Among such devices are three-dimensional and planar microelectrodes, each with their respective advantages and disadvantages. Whereas, the three-dimensional electrodes tend to allow for high fidelity recordings they only do so over a short time period (hours to days). On the other hand, the planar microelectrodes permit longer-term recordings (weeks to months), albeit at the expense of low signal resolution. Ideally, combining both advantages would permit long-term and high-resolution recordings, which, in turn, could offer new opportunities to monitor and record subtle aspects of brain activity. Inspired by the structural attributes of a synaptic cleft, our team reports here on the next generation of planar microelectrode arrays with nano-edges offering high fidelity recordings over long time periods.

## Design and analysis

**Figure 1:**
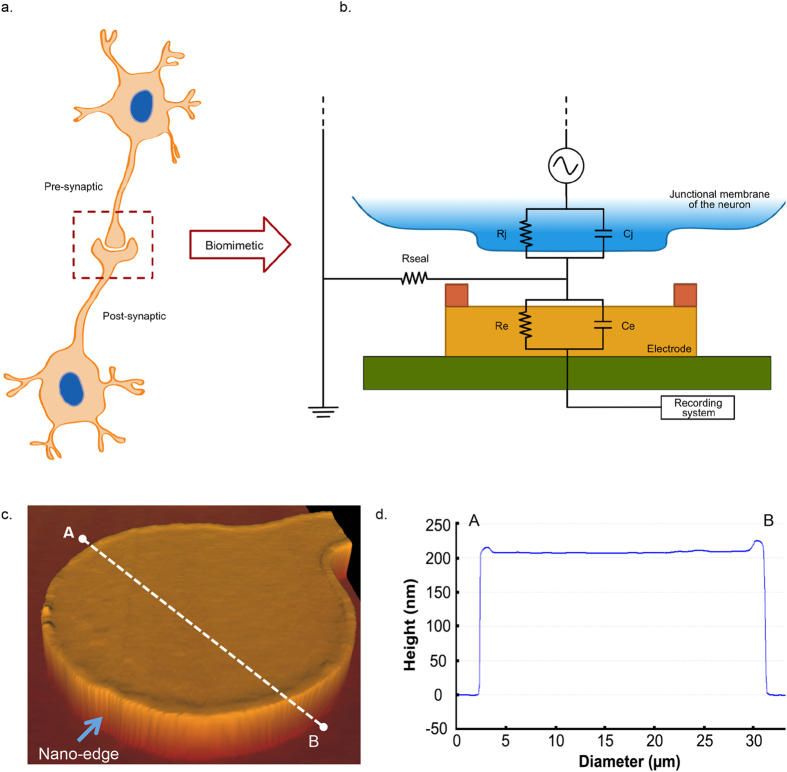
Biomimetic nano-edge microelectrode mimicking the morphological structure of a synaptic cleft. (**a)** Schematic representation of two synaptically connected neurons (the box depicts a chemical synapse between the cells). The post-synaptic terminal is shown as engulfing the pre-synaptic terminal; thereby enhancing tight physical and dielectric coupling between the neurons. (**b**) Schematic layout developed further from *Spira and Hai*^10^, illustrating an electrode-neuron interface with its analogue passive electrical circuit. Only the junctional membrane (part of the membrane in contact with the microelectrode) of the cell body is represented here (blue) – depicted to be in close contact with the electrode (yellow) and its nano-edges (orange). The non-junctional membrane (not shown in this diagram) refers to the part of the membrane not juxtaposed against the electrode. The electrode was fabricated on a silicon dioxide substrate (green) and connected to a recording system (MEA1060; Multichannel Systems, Reutlingen, Germany). The junctional membrane resistance (Rj) and conductance (Cj) are represented in parallel, similar to the electrode resistance (Re) and impedance (Ce). The sealing resistance (R_seal_) was enhanced due to the nano-edges implemented on the electrodes. (**c)** Characterization of the nano-edge microelectrodes using atomic force microscopy. A three-dimensional representation of a 30 μm microelectrode with a 40° tilt is depicted. The nano-edge is discernable around the microelectrode perimeter (blue arrow), and can be seen continuing along the connecting electrode wire (bottom right). Having the nano-edge along the wire and not limited to the circular area has the advantage that it increases the sealing resistance even when a neuron is not placed exactly on top of the microelectrode. This configuration also increased the surface area of the microelectrode that was in contact with the neuronal cell membrane (when bigger than 30 μm in diameter). (**d)** Cross-section of the microelectrode height showing the shape of the nano-edge. The microelectrodes are 30 ± 1 μm in diameter, 200 ± 15 nm in height, and the nano-edges varied between 5 and 15 nm in height and 2 to 3 μm in width. Letters ‘A’ and ‘B’ refer to the location of the cross-section taken from (**c**).

Inspired by the morphology of a synaptic cleft, whereby both pre- and postsynaptic structures are juxtaposed and semi-encapsulated, we developed microelectrodes mimicking a synapse morphology as well as neuronal juxtaposition with their adjacent cells. Specifically, microelectrodes that “bio-mimic” the postsynaptic cleft were designed to exhibit ‘nano-edges’ that provide a tighter physical and dielectrical seal between the device and the neuron. This structural geometry was also anticipated to prevent the leakage of current into the surrounding extracellular milieu, thus preserving and augmenting the functional integrity of chemical and electrical neuronal signal processing (Fig. 1a,b). We named these types of microelectrodes as “nano-edge microelectrodes”.

Gold planar microelectrodes were fabricated using a standard photolithography technique and lift-off process. Electrode sizes, and distances between them, were adjusted according to our experimental needs by modifying the photomask designs, allowing us to keep the design relatively simple, economical and scalable. Once the planar microelectrodes were fabricated, the nano-edge was added using a custom photolithography process. Following the fabrication, we characterized and validated the morphological attributes of the microelectrodes with atomic force microscopy (Fig. 1c) and were able to qualitatively confirm the presence of the nano-edges (Fig. 1d).

## Nano-edge microelectrode enables high resolution recordings

**Figure 2:**
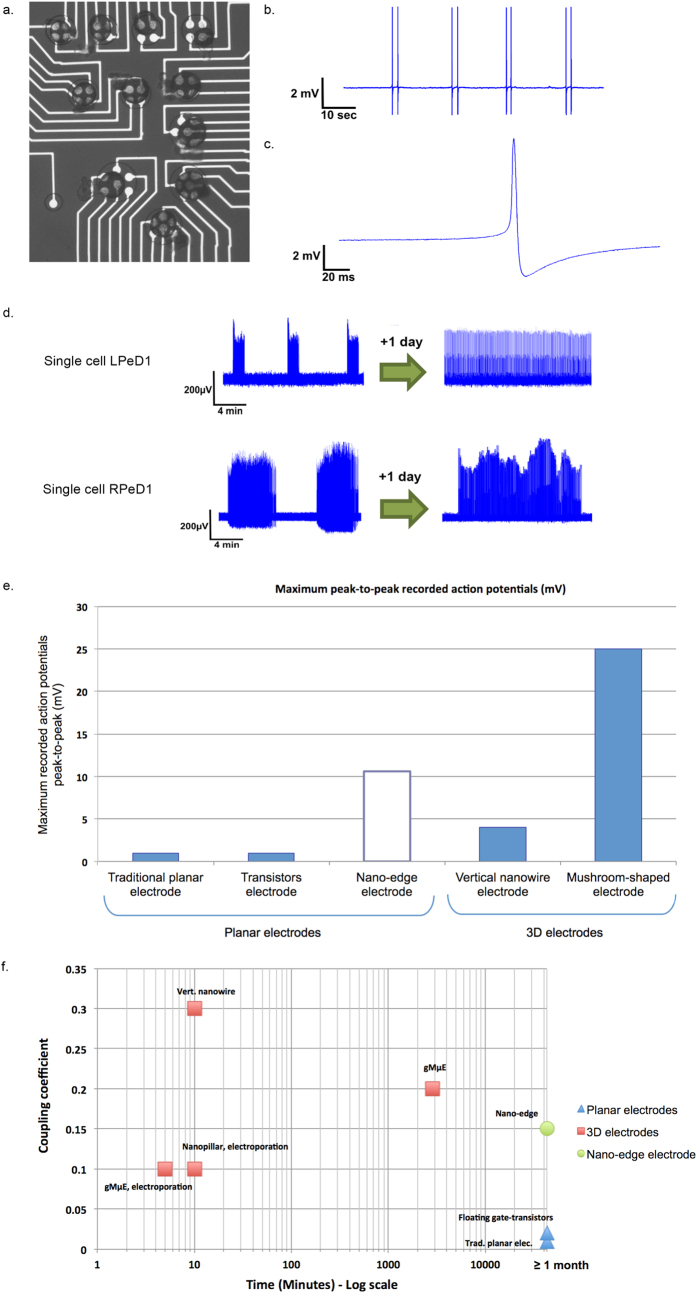
Nano-edge microelectrodes permit unprecedented resolution and long-term neural recording at the single neuron level. (**a**) Neurons were cultured on a custom designed MEA with multiple nano-edge microelectrodes grouped into clusters of 4 or 6 microelectrodes per set. The number of electrodes per set could be increased depending on the fabrication design and experimental needs. We continuously monitored neuronal activity - even if the cells had moved away from their initial culture site as described previously^23^. This setup also allows us to characterize and differentiate activity patterns from various cell types over time. An example is provided in (**d**). (**b**) Recording of action potentials from a single neuron showing distinguishable patterned activity from selected *Lymnaea* neurons^21^. (**c)** Single action potential with clearly defined depolarization followed by rebound hyperpolarization. Average of the recorded action potentials amplitude was 4.44 mV peak-to-peak (n = 13) with a maximum measured value of 10.6 mV. (**d)** Examples of distinctive spontaneous activity patterns associated to two different neurons (LPeD1 and RPeD1) resected from the mollusk *Lymnaea*. These specific activity changes recorded within identified neurons can now be studied over months and advanced drug-screening can be performed to better understand the effect of the extracellular milieu on the cells activity. (**e)** Comparison of maximum-recorded peak-to-peak action potential between the nano-edge microelectrodes compared with other types of extra-cellular electrode, showing that the nano-edge microelectrodes record higher action potentials than all other planar microelectrodes^10^,^29^, including some three-dimensional ones (e.g. vertical nanowire^7^,^8^, Mushroom shape electrode (gMμE)^5^,^9^. (**f)** Comparison of the most commonly used micro-/nano-electrodes used to record neural activity *in-vitro*. The maximum coupling coefficient and the longest reported recording time were used to evaluate electrodes capabilities. Our nano-edge microelectrodes (green circle) permit monitoring of action potentials with a coupling coefficient comparable to that of 3D electrodes (red square), and for a period of time equivalent to traditional planar microelectrodes (blue triangles).

Using the nano-edge microelectrodes, we investigated the electrical activity of single neurons (Fig. 2a). Identified neurons isolated from the mollusk *Lymnaea* were interfaced with the electrode under sterile culture conditions and spontaneous action potentials were recorded. We recorded spikes with a maximum amplitude of up to 10.6 mV peak-to-peak (n = 13; Average peak-to-peak amplitude = 4.44 mV; Min and Max range peak-to-peak amplitude = 330 μV-10.6 mV, Standard deviation = 4.08 μV) (Fig. 2b,c), which were significantly higher than those recorded through commercially available planar electrodes (typically ≤1 mV^1^). For this analysis, only those cells that completely covered at least one electrode (100%) were considered. As demonstrated by the standard deviation, the observed variability is due to numerous application specific factors. Chief among these are cell-specific variables such as the size of the neurons and the exact interfacing between their membrane and the electrode which enables the nano-edge to fully increase the sealing resistance.

**Figure 3:**
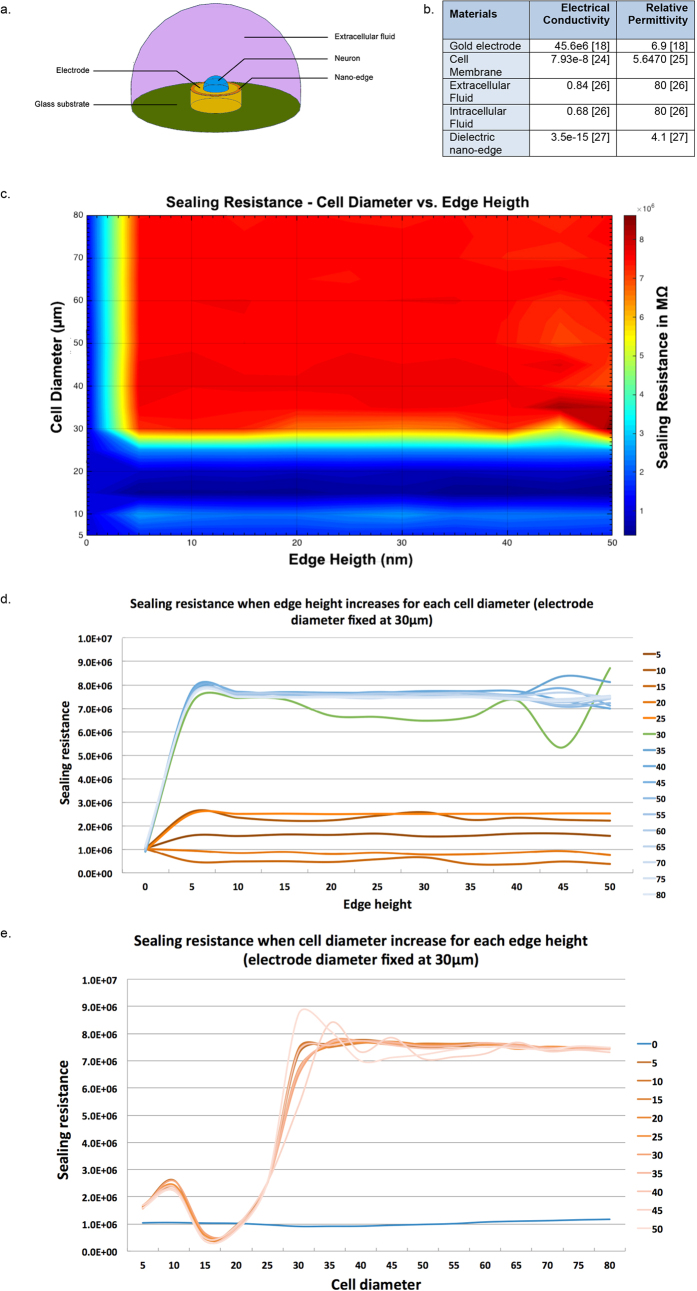
Schematic representation of the simulated elements using COMSOL Multiphysics and their physical characteristics. (**a**) Both glass substrate and extracellular fluid were modeled as infinite boundaries. The microelectrode height was set at 200 nm and its width to 30 μm as per our experimental needs. The neuron was positioned 50 nm above the microelectrodes to mimic the gap found at the neuron-electrode interfaces^10^,^16^,^17^, and was modeled with diameters in the range of 5 to 80 μm, which is representative of most vertebrate and invertebrate cell diameters. Finally, the nano-edge was modeled at various heights from 0 (no nano-edge, similar to traditional planar electrodes) to 50 nm (same height as the cleft). (**b**) Table of physical values of electrical conductivity and relative permittivity used to run the computational simulation (Refs 18, 25, 26, 27 are listed in brackets in the table). (**c)** Graphical representation of the sealing resistance disparity when computationally varying the nano-edge height and the cell’s diameter using a heat map, function of the cell’s diameter and the nano-edge height. Note the rapid increase in sealing resistance when the nano-edge is present and the cell’s diameter is equal or larger than the electrode (here 30 μm in diameter). (**d**) Variation of the sealing resistance for each cell diameter when the nano-edge increases in height. When the cell’s diameter reaches a diameter equal to or larger than the microelectrode and that an edge is present, the sealing resistance reached a plateau of 7.49 ± 0.34 MΩ. (**e**) Variation of the sealing resistance for each nano-edge height when the cell diameters increases. A dip between 10 and 25 μm can be attributed to current leakages happening when a cell’s diameter is smaller than the electrode. A similar plateau as for (**d**) can be seen.

We found the neuronal coupling coefficient to be 0.15, which is 15 times higher than what has been reported for traditional planar and resistor electrodes^10^ (0.001–0.01^1^). Thus, our novel nano-edge microelectrode recorded neural activity at a significantly higher resolution than any other traditional planar electrodes, and often better than most three-dimensional electrodes (ranging from 0.1 to 0.3) (Fig. 3e).

Moreover, three-dimensional electrodes generally monitor activity over a limited period of time (maximum 2 days using mushroom-shape electrodes^10^) due to the intrusive nature of these three-dimensional structures that compromise neuronal cell membrane and network integrity, thereby disrupting their connectivity and cellular viability. Indeed, neuronal adhesion and firm contact with the recording site are prerequisites for longer-term sustainable recordings. Neurons grown in culture tend to pull away from the recording sites due to physical tension applied by either their elongating growth cones or neurites. This, in turn, results in the neurons losing contact with the recording site, reducing the efficacy of the signal and damaging the neuronal membrane. Several approaches have been used to restrain neurons to their recording sites, albeit with limited success^11^. When three-dimensional features restrain this natural movement (e.g. spike and mushroom electrodes), neurons may eventually experience membrane rupture, cytosol leakage and cell death within a short period of time^10^.

In contrast, our planar nano-edge microelectrodes did not limit a neurons’ movement to such an extent and subtly maintained the interface with the contact site, thus enabling stable neuronal recordings for at least two weeks (recordings were conducted for a minimum of two weeks in this case and then stopped intentionally as per the experimental paradigm) but with the full potential for continued recording for another two weeks) (Fig. 3f) - like any other traditional planar electrodes^12^. Indeed, because of the presence of a nano-edge of 5 to 15 nm in height the neuronal membrane integrity was not compromised nor did the cell migrate away from the electrode. This allowed for neurons to develop networks whose activity was then continuously monitored for at least two weeks. In instances where neurons did migrate away from their initial site of electrode contact, we continued to acquire high fidelity recordings due to the following reasons: First, the adjacent nano-edge microelectrodes (grouped in multi-electrodes units of either four or six, in our case) maintained the dielectric contact with the cell. Specifically, we could use the adjacent nano-edge microelectrodes as our new monitoring site and then merge the acquired data at the end of the experiments. Secondly, to enable stable and high-resolution recordings over and above the background noise, a neuron must fully cover a typical planar electrode at all times. Any movement away from the recording site may thus render the signal undetectable. Since the signals recorded by our nano-edge microelectrodes were significantly larger, the amplitude of the action potential remained easily discernable from the noise level throughout the course of an experiment, even if the cell was not fully covering the microelectrode surface.

Taken together, we have demonstrated that our novel nano-edge design offers tremendous potential to study neural activity at the resolution of single neurons. Moreover, these electrodes also allowed us to monitor changes in the patterned neural activity (Fig. 2d), as the neuronal membrane and network properties mature over time, thus allowing us to investigate network connectivity and plasticity at a resolution never achieved before.

## Nano-edge microelectrodes functional validation using computational simulation

To validate the functional efficacy of our nano-edge microelectrodes, we ran a computational simulation. The simulation test confirmed that our observed signal enhancement could be attributed to two main factors: either an increased sealing resistance or a decreased electrode impedance. In our case, as the materials and dimensions of the microelectrode did not vary significantly from conventional planar gold electrodes^13^, a decreased impedance was an unlikely reason. Thus a change in the sealing resistance was likely the determining factor underlying higher amplitude signals. To investigate this variable, we modeled a neuron-electrode interface using the built in *Electric Currents* module in COMSOL Multiphysics (COMSOL Inc., Burlington MA). The goal of this simulation exercise was to improve on previous COMSOL models of neuron simulation^14^,^15^, and also to determine the effect of the nano-edge on the sealing resistance, which is defined as the resistance that restricts current leakage through the gap between a neuron and the electrode.

As shown in Fig. 3a, the model consists of several domains. Firstly, a glass substrate, which acts as an insulating layer, was modeled to form the basis for the MEA. The glass substrate was modeled with an infinite boundary for this simulation, meaning that the multi-physical properties were preserved along the entire surface of the glass substrate. The microelectrode was modeled above the glass using a gold cylinder with a diameter of 30 μm, which reflects the size of our microelectrodes. A thin layer of chrome was inserted in between the gold microelectrode layer and the glass, which acted as an adhesive substrate between the two layers in the fabricated devices. However, no differences in the simulation results were observed when the chrome layer was added or removed from the model. Located 50 nm above the electrode, to reflect the junctional gap in neuron-electrode interfaces^10^,^16^,^17^, the neuron was modeled using a semi-circle (ranging from 5 μm in diameter to 80 μm, to reflect the variability of cell diameters found in vertebrate as well as invertebrate models). The boundaries and volume of this semi-circle acted as the membrane and intracellular fluid, respectively. A 2 μm wide nano-edge was added to the electrode via a ring of dielectric material around its upper edges. While the nano-edge height on our fabricated microelectrode ranges between 5–15 nm, we simulated a nano-edge ranging from 0 nm (no nano-edge, similar to traditional planar electrodes) to 50 nm (height at which the nano-edge completely fills the gap between the electrode and the neuron^15^,^16^,^18^) to provide a better understanding of its effect. The remaining external space was filled with the extracellular fluid. Similar to the glass substrate, this domain of extracellular fluid was modeled as an infinite region. Figure 3b shows the values of electrical conductivity and relative permittivity used for the various materials.

Two specific meshes were used in the computational model to improve the outcomes and analysis. A standard free tetrahedral mesh was used for the neuron and the surrounding extracellular fluid. However, the free tetrahedral mesh was unable to mesh the smaller portions of the simulation due to computational limitations with regards to smaller elements, and therefore a free triangular swept mesh was implemented for these regions. This mesh was utilized for the glass substrate, gold electrode, and the thin layers in between the electrode and neuron. A swept mesh was found to be better for modeling thin layers and non-proportioned domain sizes by avoiding redundant mesh elements, which also decreased the computation time. The mesh contained between 253,178 and 157,401 mesh elements. Increasing the mesh from 250,513 to 779,642 elements resulted in a very small change of 0.02 MΩ to the sealing resistance, indicating that a larger mesh did not have an extensive impact on the results and a smaller mesh was therefore used to reduce computational time^19^. The sealing resistances calculated using this model for a nano-edge microelectrode of 30 μm in diameter ranged from 0.66 MΩ to 8.71 MΩ, depending on the height of the nano-edge and the size of the neuron simulated. When analyzing the sealing resistance values of planar microelectrodes with no nano-edge, our results were in the same range to that of *Cohen et al*.^20^ when using transistor planar electrodes, which validated the accuracy of our simulation and gave us confidence in our nano-edge simulation results.

Results of this simulation help draw two important conclusions: (1) The neuronal diameter is a determining factor underlying the sealing resistance value, which significantly increases as the diameter is equal to or larger than the circular microelectrode diameter (30 μm in our case). When a cell’s diameter is smaller than the electrode, the sealing resistance values tend to vary due to current leakage. (2) As soon as the nano-edge is present (nano-edge ≥5 nm in height) and that the neuronal diameter is equal to or larger than the electrode, the sealing resistance remains approximately the same, with an average of 7.49 ± 0.34 MΩ (standard deviation), no matter the height of the nano-edge. In the absence of the nano-edge, no significant difference in sealing resistance is observed (average of 1.03 ± 0.08 MΩ), no matter what the cell diameter is. These results are represented in Fig. 3c–e.

## Discussion

We have demonstrated that designing neuro-electronic devices that are based on natural cellular architecture could pay large dividends in the design and implementation of future bionic hybrids and drug screening devices. Specifically, simple nano-scale structural modifications can significantly increase not only the cell-coupling coefficient (by a factor of 15 in our case), but also facilitate long-term recordings at the resolution of single neurons. By exploiting parameters such as the size and spatial pattern of the microelectrodes, or the types and thickness of materials, we have optimized the recording potential of our custom designed MEAs while maintaining a relatively simple, scalable fabrication process. In addition, our computational simulation model revealed the reasons behind the nano-edge effect; an increase in the sealing resistance (R_seal_) value is observed if the cell’s diameter is at least equal to the electrode’s diameter and only if there is a nano-edge of a particular height.

### Advantages and applications

The nano-edge microelectrodes fill a large technological gap and provide a breakthrough in the field of neural recording by coupling the advantages of both planar and three-dimensional electrodes. It is now possible to monitor neuronal activity from single cells and large ensembles by maintaining stable recordings over extended time periods (weeks to months). Such attributes when combined together are necessary as they open up new opportunities to investigate advanced neural phenomena such as network formation, maturation, plasticity and dysfunction^21^. Such high fidelity recording capability may even allow us in the future to monitor sub-threshold synaptic events and distinguish between various spike patterns of bursting or tonically active neurons embedded in a complex network.

### Limitations and future development

The nano-edge microelectrodes presented here were coupled with large invertebrate neurons, which can be manipulated with ease. Broadening the scope of this technology for mammalian neurons would require reconfiguration of the electrode design, which is relatively straightforward based on the fabrication process. Although giant invertebrate neurons (40 to 80 μm diameter) generate large electrical events as compared with their vertebrate counterparts (6 to 15 μm on average), we are confident that our ability to increase the sealing resistance and therefore the amplitude of the recorded neural signals will enable higher resolution signals for mammalian cells.

Several modifications could be made in the future to improve the recording resolution. For example, using conductive materials for the nano-edge; replacing the gold electrode with another metal or alloy coated with platinum black^22^ or increasing the general roughness of the electrode surface may all enhance the quality of the recordings further. Regardless of the modifications, we believe that the nano-edge will define the future of biocompatible microelectrode arrays permitting long-term recordings at a resolution close to intra-cellular recordings.

## Methods

### Device fabrication

Devices were fabricated using a two-mask photolithography process on 49 × 49 mm, 1mm thick glass. The electrodes were sputter coated ~200 nm gold on top of a 10 nm chromium adhesion layer. Once the planar microelectrodes were fabricated, the nano-edge was added using a custom photolithography process. An epoxy based 5 μm photoresist (SU8) was then added with photolithography to provide an insulation layer over the microelectrode traces. Openings in the SU8 layer left the main microelectrode arrays bare for stimulation/recording. Sizes and intervals between microelectrodes can be adjusted according to experimental needs by modifying the photomask designs, resulting in the multi-electrodes units used in this paper shown in Fig. 2a. This represents a standard photolithography process that can be adapted to many needs.

### Cell culture

#### Snail

We used the freshwater snail *Lymnaea stagnalis* to study neuronal properties and intrinsic patterned activity. This invertebrate model provides structurally and functionally well-characterized individual neurons, which are 50–80 μm in diameter, and allow manipulation on MEAs with ease at the single cell level^23^. We used functionally well-defined and characterized neurons to study their electrophysiological activity over time.

#### Culture

Neurons were isolated and cultured as described in Syed, *et al*.^24^. Briefly, the dissected central ring ganglia from 1 to 2 month old animals were enzymatically digested with trypsin (2 mg/mL; T- 4665; Sigma-Aldrich, St Louis, MO, USA) in order to loosen the surrounding connective sheath. Trypsin inhibitor (2 mg/mL; T-9003; Sigma-Aldrich) was applied for 15 minutes to stop the enzymatic reaction. Identified pre- and post-synaptic neurons, VD4, LPeD1 and RPeD1, were isolated respectively, by gentle suction applied through a fire- polished, Sigmacote^®^-treated glass pipette (SL2; Sigma- Aldrich). The cells were then plated on the multi-electrode units of a poly-L-lysine coated MEA and maintained in brain-conditioned media (CM) prepared as described previously^21^. The neurons were allowed to settle overnight and used for experiments 12–18 hours post-culture.

### Biocompatibility study

To assess the biocompatibility of our MEAs, we considered three key aspects; whether the neurons (a) maintain their intrinsic membrane properties, (b) were capable of growth and (c) developed synaptic connections.

First, we tested whether neurons cultured on our MEAs maintained their intrinsic membrane properties and were able to maintain a voltage differential across their membrane and fire action potentials. All cells (100%, n = 20) were consistently able to do so at least 10 days post-culture.

Second, to determine whether neurons cultured on our MEAs exhibited growth, cells were plated on the MEA and allowed to extend neurites. We found that all cells (n = 9) exhibited extensive outgrowth for cultured neurons to grow at a rate of up to 1 mm per 24 hours, indicating a high degree of biocompatibility with the substrate materials used. Neurons remained viable for at least a month, similar to what is seen on conventional coated glass coverslips, as long as the extracellular environment is regularly replaced with new solution.

Finally, we determined whether synapses could be formed on our MEA. When pre- and post-synaptic neurons (VD4 and LPeD1, respectively) were cultured together in a soma-soma configuration, action potentials could be triggered in VD4 using intracellular sharp electrodes which elicited 1:1 EPSPs of constant amplitude and latency, as seen when cultured on our SS-Chip (95% of paired cells formed a synapse within 24 hours on the SS-Chip, n = 20).

This high degree of biocompatibility was expected as we used materials that have been previously reported as safe in the literature^23^,^28^.

## Additional Information

**How to cite this article**: Wijdenes, P. *et al*. A novel bio-mimicking, planar nano-edge microelectrode enables enhanced long-term neural recording. *Sci. Rep.* **6**, 34553; doi: 10.1038/srep34553 (2016).
